# Notes and News

**Published:** 1894-04-28

**Authors:** 


### Notes and News.

Collected and Communicated.

MEETINGS OF THE WEEK.

Metropolitan Hospital, Kingsland Road, Festival Dinner, Hôtel Metropole, Monday, April 30th.

Children's Hospital, Great Ormond Street, Festival Dinner, Hôtel Metropole, Saturday, May 5th.

H.R.H. the Duchess of Albany will present certificates to lady students at the Sanitary Institute, Margaret Street, on May 10th. The ceremony is fixed for three o'clock. The Duchess takes great interest in the institute, and was present herself at some of the lectures on "Modern Hygiene," given by Professor Schofield, in connection with which the diplomas are being given.

A largely attended public meeting was held in the Board Schools, Quarry Hill, Grays, on Tuesday, the 24th inst., at 7.30 p.m., under the presidency of Mr. E. R. Parker, J.P., to consider what steps should be taken in connection with the offer of Mr. J. Passmore Edwards to build a Cottage Hospital in that neighbourhood. Much difference of opinion existed at the meeting as to the site of the hospital. Grays and Tilbury are both at present without hospital accommodation, and though within a mile and a-half of one another, there is little or no communication after dark, in consequence of the swamp that exists between the two places. The surrounding neighbourhood is exceedingly poor, and could with difficulty, maintain one hospital, and where this is to be placed is the question. In the end a committee was formed to inquire into the possibilities of obtaining adequate support to maintain a small hospital, and to report as to the best site, providing it is found that sufficient funds will be forthcoming. A vote of thanks to Mr. Passmore Edwards for his generous offer concluded the meeting.

If all sanitary authorities showed the enterprise and common sense displayed by the Chelmsford Board, not only should we hear fewer complaints as to the quantity and quality of the water supply in rural districts, but typhoid would, doubtless, also be far less prevalent. At Danbury, a village in the district, water was abundant, whilst in near lying hamlets, in dry seasons, water was only procurable from the most doubtful sources, such as ponds and ditches. The surveyor, Mr. J. Smith, and Dr. Thresh, the medical officer, conceived the idea of carrying out a scheme of supplying neighbouring villages with the overflow of water at Danbury by the simple means of gravitation. Four parishes have accordingly been supplied with good water, the initial outlay being met by a loan of £3,000 from the Local Government Board. The annual expenditure will be most trivial, whilst the inhabitants of the villages will no longer be troubled with scarcity of water.

We are informed that the Lord Mayor having kindly consented to preside at the Metropolitan Hospital dinner on April 30th next, at the Hotel Metropole, the committee are making the utmost exertions in connection with this dinner to obtain funds to pay off the pressing liabilities, and to enable them to carry on the work of the institution in an efficient manner. Situated as it is in the heart of the poorest district of the north-east of London the hospital affords relief to a population of nearly half a million inhabitants. During last year 857 in-patients were under treatment, whilst the attendances of the out-patients numbered 73,283. In the new buildings, which were opened in 1887, there is accommodation for 160 beds, but owing to lack of funds only 54 are at present available for in-patients. As this number is quite inadequate to meet the pressing demands upon them, the committee are now under the painful necessity of refusing numerous and urgent applications for admission. The committee therefore most earnestly appeal for aid to help them to continue and extend the useful work of the institution.

The result of the inquiry at the Forest Gate Schools, respecting the food supplied to the seven hundred children within its walls, could hardly be more unsatisfactory. The serious charges have not been refuted. The diet entries made by the superintendent have been shown to be totally unreliable, to say the least, yet this official retains his post, whilst the person who was instrumental in causing the exposure of the scandal is rewarded by the loss of his post. Every encouragement should be given by those interested in the welfare of the helpers, be they children or adults, to officials who will expose abuses at the risk of losing their posts, and further, the public, who are not wholly indifferent to the management of orphanages, especially which their money goes to support, should occasionally visit the institutions at meal times without giving notice of their visit, and thus by a little sacrifice of personal convenience do more to insure the proper nourishment of the children than a large expenditure of money only would in many cases secure them. Finally, in respect to orphanages supported by voluntary charity, we would be chary of encouraging those which already manage economically to seek to retrench in the expenditure of food, a course which, in examining the reports of orphan asylums, we observe is by no means uncommon.

